# A Dual‐Perspective Comparison of Classical and Clinic‐Based Multidisciplinary Team Models in Cancer Care: A Cross‐Sectional and Qualitative Study

**DOI:** 10.1002/hsr2.71787

**Published:** 2026-01-28

**Authors:** Mengying Li, Leihua Chen, Yanbing Liu, Xiaoying Jiang

**Affiliations:** ^1^ Party and Government General Office, Fudan University Shanghai Cancer Center Shanghai China; ^2^ Department of Oncology, Shanghai Medical College Fudan University Shanghai China; ^3^ Outpatient Office, Fudan University Shanghai Cancer Center Shanghai China

**Keywords:** cancer care, multidisciplinary teams, patient satisfaction, physician perspectives, tumor board

## Abstract

**Background and Aims:**

Multidisciplinary teams (MDTs) represent a key trend in global cancer care. This study aims to discuss the applicability and benefits of two MDT models in a Chinese tertiary cancer hospital, the classical MDT consultation meeting model and the MDT clinic model, regarding process and efficiency, physicians' preferences, and patients' satisfaction.

**Methods:**

We conducted a user‐side cross‐sectional survey among patients in MDT clinics (*n* = 520) and consultation meetings (*n* = 123) via convenience sampling. The survey captured basic information, treatment results, and satisfaction (5‐point Likert scale). On the provider side, semistructured interviews were conducted among 28 physicians on MDTs, focusing on the team's working mode and communication style.

**Results:**

Since 2005, the hospital has established 15 MDTs. The classical model, operating on a fixed schedule, is valued by physicians for educating young staff. Since 2020, MDT clinics in 8 disciplines have streamlined the appointment process and involved patients directly in discussions. The majority of physicians (25/28) affirmed that this model has improved the efficiency of developing diagnostic and treatment plans. The overall satisfaction of patients with the MDT clinic was (4.66 ± 0.54), which was higher than their satisfaction with the classical MDT model (4.53 ± 0.53). In the MDT clinic model, non‐local patients reported greater satisfaction (4.68 ± 0.58) than did local patients (4.65 ± 0.53), while the opposite was true of the classical MDT model. Patients with more complex tumor types (*F* = 2.35), longer wait times (*F* = 9.53), or no clear treatment plan (*t* = 9.49) reported lower satisfaction with both models (*p* < 0.05).

**Conclusion:**

For regional hubs featuring the necessary resources, the establishment of a multidisciplinary clinic model for diagnosis and treatment is strongly encouraged. The classical MDT meeting is indispensable for complex case deliberation and physician training, while the multidisciplinary clinic model can be utilized to enhance patient‐centered care and satisfaction.

## 1 Background

Cancer is a leading cause of death and disability worldwide [1]. It is estimated that China accounts for approximately one‐fourth of all new cancer cases and one‐fifth of all cancer deaths globally [2]. Due to advancements in medical technology and the complexity of tumor pathogenesis, the treatment of cancer often involves multiple disciplines, such as surgical oncology, medical oncology, radiology, and interventional therapy. The traditional model of medical decision‐making based on individual experience is no longer applicable to cancer treatment. Multidisciplinary teams (MDTs) have gradually come to represent a new trend in cancer diagnosis and treatment worldwide [3]. Several studies have suggested that MDTs are associated with changes in clinical outcomes, reduced time to treatment after diagnosis [4], higher patient satisfaction [5], and better adherence to clinical guidelines [6, 7].

The classical model of MDT has relied primarily on conference‐based discussions, which are typically held among healthcare professionals to promote discussion concerning treatment plans for hospitalized patients. In recent years, the emergence of multidisciplinary clinics has highlighted a shift from inpatient‐focused meetings to outpatient‐based approaches. The MDT clinic is designed to serve as a one‐stop shop for patients [8], who can now receive evaluations and treatment recommendations from multiple specialists during a single visit. At the international level, various hospitals and clinics, such as the University of Pittsburgh Medical Center in 2008 [9], Johns Hopkins Breast Center in 2015 [10], and Kingston Health Sciences Centre in 2016 [11], have taken the initiative to establish multidisciplinary clinics and have achieved notable success in patient care.

In China, the use of multidisciplinary treatment for cancer has been encouraged by the government and healthcare organizations [12]. The National Health Commission of China issued the “Notice on the Pilot Work of Multidisciplinary Diagnosis and Treatment of Cancer” in August 2018 [13], which officially proposed to perform pilot work for the multidisciplinary diagnosis and treatment of cancer nationwide. Fudan University Shanghai Cancer Center (FUSCC) in southeast China, which was one of the first pilot hospitals in multidisciplinary oncology, has implemented these two types of MDT models since 2005 and 2019, respectively. Notably, at a time when MDT clinics had not yet been widely adopted in China, this hospital was among the earliest to establish MDTs and MDT outpatient services [14].

A key distinction in the care delivery process between the traditional conference‐based model and the newer one‐stop MDT clinic model necessitates an evaluation beyond clinical outcomes. Given such differences, the patient and provider experience within these MDT structures is critical, as it can directly affect implementation success and model acceptance. Research indicates that patient satisfaction is influenced by factors such as direct involvement in discussions and the clarity of communication received from the team [15]. Concurrently, physicians' preferences for a particular MDT model are shaped by its perceived efficiency, impact on their professional development, and the quality of interdisciplinary collaboration [16]. However, a direct comparison of these two prevalent MDT models, especially from the dual perspectives of both physicians and patients within the same high‐volume institution, remains underexplored in the literature. Therefore, the aim of this study was to compare the classical MDT meeting and the MDT clinic model regarding their operational process, efficiency, physicians' preferences, and patients' satisfaction. By conducting this study, we hope to obtain a deeper understanding of the applicability and strengths of each model and to identify the most effective way to provide comprehensive cancer care.

## 2 Methods

**Figure 1:**
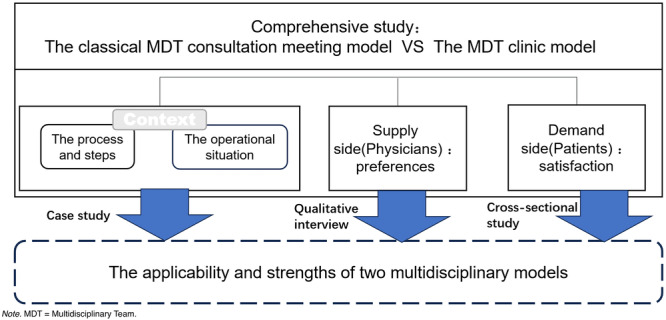
Schematic overview of the study design for comparing the classical MDT meeting model and the MDT clinic model. The research framework integrated multiple components to conduct a dual‐perspective comparison. Analysis of the Context (processes, steps, and operational situation) provided the foundation. Insights were gathered from the Supply side (physicians via qualitative interviews) and the Demand side (patients via a cross‐sectional survey). These streams of evidence converged in a case study aimed at evaluating the applicability and strengths of the two MDT models.

The research methodology was designed to explore both the provider and user perspectives of the MDT model. This study was conducted at FUSCC, Pudong Campus, a leading tertiary cancer hospital in China. The newly established Pudong Campus, which opened in 2019 and has an approved inpatient bed capacity of 799, is specifically designed to support integrated care models such as the MDT clinic. A key feature of this campus is its dedicated physical zone for multidisciplinary clinics, which capitalizes on the new facility's ample space to support the collaborative workflow required for MDT‐based care. We initiated by collecting data from the hospital's MDT information system, including the annual number of MDT clinic visits and MDT meeting discussions conducted. These data were utilized to summarize the establishment process of the hospital's MDT teams and their operational status in recent years. Furthermore, we conducted interviews with physicians in the MDTs, serving as the providers within the model. These interviews aimed to gain insights into their routine work patterns and preferences between the two models. Turning to the user side of the model, we conducted individual service satisfaction surveys targeted at patients in MDT clinics and consultation meetings. This survey focused on gathering patients' basic information, details of their diagnosis and treatment outcomes, as well as their satisfaction levels. A schematic diagram of the study design is shown in Figure 1.

### 2.1 Cross‐Sectional Study

Sampling method: A questionnaire survey was conducted among patients in MDT clinics and consultation meetings via convenience sampling. The primary inclusion criterion for inclusion was the patient's willingness to provide written informed consent and complete the survey. Consequently, a total of 532 and 129 questionnaires were administered to patients in MDT clinics and consultation meetings, respectively. Questionnaires returned incomplete were deemed invalid and excluded from the analysis. This resulted in 520 and 123 valid questionnaires being returned, for effective recovery rates of 97.7% and 95.3%.

Questionnaire development: Due to the lack of validated, multidisciplinary‐specific questionnaires for reference, we developed our own questionnaire based on the patient satisfaction scales developed by Sidhartha Satpathy [17] and Huigang Liang [18]. Before survey distribution, a small‐scale validation was conducted with 20 patients to ensure that the questions were unambiguous, that the duration of the survey was acceptable, and that no privacy violations occurred. The questionnaire consisted of two parts and is included in the Supporting material. The first part collected basic information regarding the patient and the MDT clinic, including the demographic information of the patient, the type of MDT clinic or meeting, the length of time from appointment to actual visit, and whether the patient received a clear diagnosis and treatment plan after the visit. The second part evaluated patients' satisfaction with the experience at the MDT clinics or meetings in terms of five dimensions: the convenience of registration, the hospital environment, the service attitudes of clinicians, the benefits of MDT decision‐making, and the rationality of the recommendations. Each dimension of satisfaction was scored on a 5‐point Likert scale, in which responses of “strongly disagree,” “disagree,” “generally,” “agree,” and “strongly agree” were assigned scores ranging from 1 to 5 points, respectively.

Data analysis: Stata 16.0 software was used to support the statistical analysis of the quantitative data. Descriptive statistics were applied to summarize the basic demographic information of the sample. The primary, prespecified analyses adopted the *t*‐test to compare the overall satisfaction scores between the two models, as well as the scores across five specific dimensions under each model. Exploratory analyses, including subgroup comparisons of factors influencing satisfaction, employed *t*‐tests for data grouped into two categories and analysis of variance (ANOVA) for multiple groups. All statistical tests were two‐sided, and *p* < 0.05 was considered to indicate statistical significance.

### 2.2 Qualitative Interviews

Semistructured interviews were conducted with physicians on the MDTs that participated in these two MDT models. To be eligible for the interviews, physicians were required to have practical experience with both the classical MDT meeting model and the MDT clinic model, with at least 1 year of involvement in the latter. We sent interview invitations to all eligible physicians in our hospital and received positive responses from those who provided informed consent and were available during the study period. In total, 28 physicians, including 24 core team members and 4 extension team members from 7 MDTs, participated in the interviews. The interviews focused on comparing the two models in efficiency and impact both on physicians and patients, as well as exploring aspects such as teamwork patterns and communication methods. The interview data were anonymized, and thematic analysis was employed for manual coding and theme extraction. The interview outline is shown in the Supporting Information.

### 2.3 Ethical Considerations

The study protocol was reviewed and granted an exemption from ethical review by the Shanghai Cancer Center Institutional Review Board (Confirmation Letter No. 2409‐No.003). All participants, including both patients and physicians, provided written informed consent before taking part in the study.

## 3 Results

### 3.1 Context

To improve the standardization of cancer diagnosis and treatment comprehensively, FUSCC established 15 MDTs since 2005. Operating under a “three‐fixed” model (fixed time, venue, and personnel), these teams convene doctors from various disciplines to discuss challenging cases without direct patient involvement (Supporting Information: Figure S1). In May 2020, the hospital launched MDT clinics at its Pudong Campus. This model, depicted in Supporting Information: Figure S2, brings doctors and patients together in the same consulting room for collaborative discussion. This approach reduces repeat visits across different departments and provides greater convenience for patients.

### 3.2 The Process and Steps Involved in Both Models

The processes involved in establishing expert teams to support the two multidisciplinary treatment models involved similar steps. Each MDT was first assigned a chief expert. The chief expert was responsible for chairing discussions and finalizing treatment plans. The chief expert further selected members from the departments of surgical oncology, radiation oncology, medical oncology, and various diagnostic specialties (imaging, pathology, nuclear medicine, etc.) to form a team and ensures that the team members are relatively fixed for each discussion.

**Figure 2:**
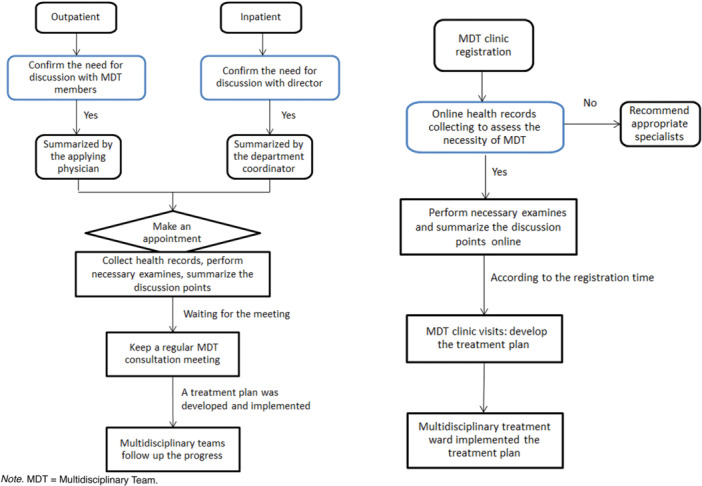
Flowchart comparing the procedural steps of the classical MDT meeting model and the MDT clinic model. The left and right pathways depict the distinct workflows of the classical MDT meeting and the MDT clinic, respectively. Key differences include the method of case preparation, the scheduling process, and the integration of patient involvement during the discussion. Both models conclude with the implementation and follow‐up of a multidisciplinary treatment plan.

The two multidisciplinary diagnosis and treatment models differed significantly in terms of the approaches they took to the consultation process (Figure 2). The classical MDT consultation meeting was held once per week at a fixed time and place. Before the meeting, the health records of patients were collected by the coordinators of each department in advance and submitted to the secretariat to make the assignments. Patient information was summarized and reported on the day of the MDT discussion. In contrast to classical MDT meetings, the MDT clinic did not require patients to schedule and wait for an expert panel meeting; instead, patients could independently select an MDT team at the time of initial registration. Furthermore, the MDT clinic features a corresponding multidisciplinary treatment ward, which involves clinicians from multiple disciplines working in the same ward to ensure that the entire process of disease diagnosis and treatment is multidisciplinary in nature (Supporting Information: Figure S3).

### 3.3 The Operational Situation

As of 2022, the hospital had established MDT clinic teams led by 10 professors across 8 specialties, including breast surgery, pancreatic surgery, and gynecology (Table S1). The total number of patients served by MDT clinics had exceeded 10,000 since their launch in 2020. The data concerning patients with breast tumors were further analyzed as an example, as shown in Supporting Information: Table S2. In 2021, a total of 88 patients were discussed in classical MDT consultation meetings, whereas the number of MDT clinic outpatient visits reached 1237. In terms of the proportion of nonhospitalized patients, the rate for the MDT clinic model (75.56%) was higher than that for the classical MDT meeting model (50.00%). Among hospitalized patients, the average total hospital stay and preoperative hospital stay durations for the classical MDT meeting model (7.18 and 4.18 days, respectively) were longer than those for the MDT clinic model (5.94 and 3.60 days, respectively).

### 3.4 Physicians' Preferences

The analysis resulted in 37 codes that were grouped into three themes: communication and collaboration, efficiency, and impact on patient comfort and outcomes. Table 1 (Sections ii–v) presents selected quotations corresponding to each identified theme. The communication and collaboration theme describes interactions between physicians and patients. A key finding from the interviews was that respondents recognized the value of working in a multidisciplinary setting, particularly for younger physicians. The majority of physicians interviewed discussed the value of having access to a diverse range of expertise, thus facilitating a more comprehensive and holistic approach to patient care. However, a notable finding pertained to the degree of deference displayed by some physicians towards the diagnostic opinion of the chief expert on the team. Of the 28 physicians interviewed, 6 reported hesitation about challenging the chief physician's diagnostic or treatment recommendations. This finding suggests that while the multidisciplinary setting promotes collaboration, certain hierarchical barriers may still limit open discussion and criticism.

**Table 1:** Analysis of themes and examples from physicians interview.

Theme	Quotation
**Communication and collaboration**	
For both models	It provides us with a platform for mutual learning. Being able to collaborate and discuss cases with specialists from different fields enhances our understanding of the patient's condition and broadens our perspectives on possible treatment options.
MDT meeting	Preparing for these discussions is indeed a rigorous process. But these discussions provide an opportunity for young physicians to engage with their peers and senior colleagues, learning from their perspectives and experiences.
MDT clinic	There's a certain hierarchy, and the chief expert's opinion usually carries a lot of weight. However, I do believe that open discussions and constructive feedback are essential for the best patient care.
**Efficiency**	
Comparison between the two models	In the past, I would have had to wait for a week or longer to get feedback from the oncologist on whether the patient was suitable for chemotherapy. But with the MDT clinic, we were able to discuss the case together in real time, and the decision‐making process was much faster and more efficient. It not only improved the patient's outcome but also saved us a lot of time and effort.
MDT clinic	One significant advantage is when patients and their families are directly involved in the discussion in our multidisciplinary clinics. We can inquire about their acceptance and financial status while formulating a treatment plan. This direct communication enhances efficiency, as we can tailor the plan to their specific needs and circumstances.
**Impact on patient comfort and outcomes**	
For both models	Patients often express a high level of confidence and satisfaction with the treatment plans formulated in the presence of so many experts.
MDT clinic	As a specialized hospital serving the southeastern region, our hospital attracts many patients from outside the area, who come here seeking our expertise. Many of these patients have undergone initial screenings at their local hospitals but have not received a definitive diagnosis or treatment plan. In such cases, referring them to our MDT clinic for an initial consultation is highly beneficial.
Comparison between the two models	For some routine tumor types with standardized treatment approaches, the classical discussion can indeed be sufficient. However, for more complex malignancies like pancreatic cancer, I believe that it is essential to involve a multidisciplinary clinic from the very beginning of the diagnostic process. The early involvement of specialists from different fields can lead to a more holistic evaluation, faster diagnosis, and ultimately, better outcomes for the patient.

Abbreviation: MDT, multidisciplinary team.

In terms of the efficiency of the two multidisciplinary care models, nearly all physicians (25/28) reported that the MDT clinic model reduced the time required to diagnose patients and formulate treatment plans compared to the classical meeting discussion model. This increase in efficiency was attributed to several factors. First, the MDT clinic setting enabled direct access to patients and their families, eliminating delays associated with information relay between different specialists. Second, the MDT clinic model facilitated a more patient‐centered approach. By involving patients and their families directly in the discussion, physicians could assess the patient's willingness and ability to adhere to the recommended treatment plan, while accounting for factors such as treatment acceptance and financial constraints. This approach not only improved efficiency but also ensured that the treatment plan was tailored to the patient's individual needs and preferences.

In discussions of the applicability of the two models, varying opinions were expressed. Some physicians noted that for common tumor types with standardized treatment pathways, the classical MDT meeting model was adequate. Others underscored the value of the MDT clinic model in cases involving more complex tumor types, such as pancreatic cancer. They emphasized the need for the early application of this model at the diagnostic stage through direct patient‐team communication. Additionally, some physicians expressed confidence in the future expansion of MDT clinics to more regional medical hubs.

### 3.5 Patient Satisfaction

Data were collected from 520 MDT clinic patients and 123 patients participating in MDT consultation meetings to support this cross‐sectional survey, with most respondents being non‐local patients (417, 64.85%). Basic respondent demographic characteristics are presented in Table 2. The sample included slightly more female patients than male patients, with female patients accounting for 58.48% of the total sample. The majority of patients were aged 45–60 years (220, 34.21%), followed by patients aged 35–45 years (194, 30.17%). The majority of patients were married (538, 83.67%). Most patients had a bachelor's degree or junior college education (264, 41.06%). The respondents were recruited from a total of 11 MDT clinics and 5 MDTs, which mainly focused on neuroendocrine tumors (162, 25.19%), pancreatic tumors (106, 16.49%), and gynecological oncology (97, 15.09%). MDT teams with fewer than 20 respondents were uniformly categorized as “other.” The length of time from appointment to actual visit was mostly equal to or less than 3 days (221, 42.50%) in MDT clinics, while MDT meetings often require more than 7 days (65, 52.80%). A total of 93.31% of the patients reported receiving a clear diagnosis and treatment plan.

**Table 2:** Basic information of respondents.

Variables		MDT meeting *n* (%)	MDT clinic *n* (%)	Sum *n* (%)
Gender	Male	51 (41.46)	216 (41.54)	267 (41.52)
	Female	72 (58.54)	304 (58.46)	376 (58.48)
Marriage	Unmarried	5 (4.07)	59 (11.35)	64 (9.95)
	In marriage	109 (88.62)	429 (82.50)	538 (83.67)
	Widowed or divorced	9 (7.32)	32 (6.15)	41 (6.38)
Education degree	Junior high school and below	44 (35.77)	120 (23.08)	164 (25.51)
	High school or technical secondary school	25 (20.30)	120 (23.08)	145 (22.55)
	Bachelor's degree or college degree	40 (32.52)	224 (43.00)	264 (41.06)
	Postgraduate or above	14 (11.38)	56 (10.77)	70 (10.89)
Age	≤ 35	22 (17.89)	134 (25.77)	156 (24.26)
	36–45	35 (28.46)	159 (30.58)	194 (30.17)
	46–59	50 (40.65)	170 (32.69)	220 (34.21)
	≥ 60	16 (13.01)	57 (10.96)	73 (11.35)
Residency status	Local	49 (39.84)	177 (34.03)	226 (35.15)
	Nonlocal	74 (60.16)	343 (65.97)	417 (64.85)
Tumor type	Pancreatic tumor	3 (2.44)	103 (19.81)	106 (16.49)
	Breast tumor	29 (23.58)	65 (12.50)	94 (14.62)
	Colorectal tumor	39 (31.71)	21 (4.04)	60 (9.33)
	Gynecological tumor	8 (6.50)	89 (17.12)	97 (15.09)
	Neuroendocrine tumor	—	162 (31.15)	162 (25.19)
	Hepatobiliary tumor	44 (35.77)	28 (5.38)	72 (11.20)
	Lung cancer with brain metastasis	—	24 (4.62)	24 (3.73)
	Other	—	28 (5.38)	28 (4.35)
Average length from appointment to actual visit	0–3 days	24 (19.50)	221 (42.50)	245 (38.10)
	4–7 days	34 (27.64)	165 (31.73)	199 (30.95)
	> 7 days	65 (52.80)	134 (25.77)	199 (30.95)
Whether a clear treatment plan was obtained	Yes	117 (95.12)	483 (92.88)	600 (93.31)
	No	6 (4.88)	37 (7.12)	43 (6.69)

Abbreviation: MDT, multidisciplinary team.

The satisfaction evaluation results are shown in Table 3. Patients' overall satisfaction score regarding the MDT clinic was 4.66 ± 0.54, which was higher than the classical MDT model score of 4.53 ± 0.53. Among the five dimensions included in the survey, patients assigned the highest ratings to physicians' service attitudes. A further analysis was conducted to explore the impacts of different factors on satisfaction scores (Table 4). In terms of demographic factors, gender, education level, and residency status had significant effects on satisfaction. Patients with higher education levels and female patients reported lower satisfaction than male patients across all five dimensions. In line with the MDT clinic model, nonlocal patients reported greater satisfaction than did those who resided in Shanghai, while the opposite was true with regard to the classical MDT model. In terms of diagnostic factors, tumor type, number of days spent waiting, and receipt of a clear diagnostic plan significantly influenced patient satisfaction. Patients with more complex tumor types, longer wait times, or no clear treatment plan reported lower satisfaction with both models.

**Table 3:** Satisfaction scores of respondents.

Satisfaction score	MDT meeting		MDT clinic		*t*	*p* value
	*x̅*	*σ*	*x̅*	*σ*		
Overall	4.540	0.53	4.66	0.54	2.55	0.006
Convenience of registration	4.520	0.03	4.63	0.06	2.36	0.009
Hospital environment	4.634	0.62	4.72	0.68	−0.11	0.54
Service attitude of clinicians	4.680	0.05	4.75	0.03	1.51	0.07
Benefits of MDT decision‐making	4.523	0.05	4.73	0.03	2.15	0.02
Rationality of the charge	4.390	0.06	4.48	0.04	1.30	0.10

Abbrevaition: MDT, multidisciplinary team.

**Table 4:** Influencing factors of satisfaction.

Variables			Overall satisfaction scores (*x̅ *± *σ*)	*F/t*	*p* value	MDT meeting (*x̅ *± *σ*)	MDT clinic (*x̅* ± *σ*)
Demographic factors	Gender	Male	4.73 ± 0.41	3.82	< 0.001	4.66 ± 0.42	4.75 ± 0.03
		Female	4.57 ± 0.59			4.45 ± 0.47	4.60 ± 0.04
	Age	≤ 35	4.55 ± 0.55	1.00	0.39	4.47 ± 0.54	4.56 ± 0.68
		36–45	4.64 ± 0.52			4.52 ± 0.49	4.66 ± 0.56
		46–59	4.68 ± 0.45			4.55 ± 0.51	4.74 ± 0.40
		≥ 60	4.59 ± 0.53			4.54 ± 0.44	4.67 ± 0.45
	Education degree	Junior high school and below	4.64 ± 0.50	5.14	0.002	4.52 ± 0.44	4.69 ± 0.52
		High school or technical secondary school	4.70 ± 0.42			4.65 ± 0.35	4.72 ± 0.44
		Bachelor's degree or college degree	4.65 ± 0.53			4.55 ± 0.49	4.67 ± 0.54
		Postgraduate or above	4.41 ± 0.69			4.37 ± 0.60	4.43 ± 0.71
	Residency status	Local	4.64 ± 0.40	1.78	0.038	4.56 ± 0.42	4.65 ± 0.53
		Non‐local	4.60 ± 0.55			4.51 ± 0.52	4.68 ± 0.58
Diagnostic factors	Tumor type	Hepatobiliary & Pancreatic tumor	4.67 ± 0.41	2.35	0.003	4.56 ± 0.40	4.69 ± 0.46
		Colorectal tumor	4.64 ± 0.43			4.54 ± 0.46	4.69 ± 0.57
		Gynecological tumor	4.61 ± 0.55			4.54 ± 0.41	4.64 ± 0.59
		Other	4.50 ± 0.66			4.51 ± 0.53	4.39 ± 0.46
	Average length from appointment to actual visit	0–3 days	4.70 ± 0.53	9.53	< 0.001	4.64 ± 0.51	4.71 ± 0.55
		4–7 days	4.62 ± 0.54			4.58 ± 0.42	4.64 ± 0.56
		> 7 days	4.52 ± 0.49			4.34 ± 0.47	4.63 ± 0.51
	Whether a clear treatment plan was obtained	Yes	4.69 ± 0.45	9.49	< 0.001	4.56 ± 0.39	4.72 ± 0.45
		No	3.94 ± 0.90			4.07 ± 0.77	3.92 ± 0.94

Abbrevaition: MDT, multidisciplinary team.

## 4 Discussion

Our study provides a direct comparative analysis of the classical MDT meeting and the MDT clinic model from the dual perspectives of physicians and patients at FUSCC, a national MDT pioneer in China since 2005. Building on this foundational experience, our study leveraged a unique setting to address a critical literature gap by offering empirical insights from a high‐volume cancer center into the comparative strengths of two prevalent MDT care delivery structures. Our findings demonstrated that both models have positive potential to foster professional development among physicians and facilitate the development of patient diagnosis and treatment plans. Previous studies and clinical practice have shown that MDTs significantly improve clinical decision‐making [19, 20], reduce time to treatment [21], and improve patients' treatment compliance [22]. We also found that the MDT outpatient model had a particularly significant impact on improving patient satisfaction. Our results were consistent with those reported by Kedia regarding patients and their caregivers in the context of the MDT clinic model [23], which revealed that this model could improve physician collaboration and patient convenience while reducing patient confusion and anxiety.

From a user‐side perspective, patients reported high satisfaction with both models, with higher scores for the MDT clinic. This difference may be attributed to several factors. First, patients in the MDT clinic were able to participate more actively and realistically in discussions with the MDT of physicians [5, 24]. This interaction fostered a sense of comfort and trust, as patients understood their condition was undergoing a comprehensive evaluation. As a result, patients felt more empowered to share their true feelings and opinions, leading to a more holistic understanding of their condition [25]. This understanding, in turn, facilitated faster decision‐making and treatment planning. Second, it is noteworthy that non‐local patients expressed higher satisfaction with the MDT clinic model than did local patients. This finding indicates that the model drew patients from surrounding regions seeking more specialized, comprehensive care. The multidisciplinary clinic, therefore, not only serves the local population but also acts as a referral center for more complex cases. Moreover, a significant factor influencing patient satisfaction was the availability of a definitive treatment plan during the diagnosis and treatment process. This finding highlights the importance of efficiency with regard to the finalization of treatment plans within the multidisciplinary framework. Patients value certainty and a clear understanding of their treatment options, a clarity that is often lacking in traditional conference‐based discussions, where decisions tend to take longer to finalize.

As the only individuals in this study to have experienced both models, physicians offer a unique perspective, thus facilitating a direct comparison between the two models. They strongly endorsed the value of the multidisciplinary environment for both professional development and the advancement of clinical and research practices. In particular, they emphasized the role of the classical MDT meeting model in the promotion and growth of young physicians, as it encourages clinicians to think beyond their comfort zones and consider alternative perspectives [26, 27]. However, while the exchange of ideas and collaboration among specialists are valuable, it can often be time‐consuming. Physicians may have to wait for their turn to speak, and these discussions can sometimes be hindered by technical details or theoretical debates [28, 29]. In contrast, physicians praised the clinic model for its efficiency and structured workflow, facilitating real‐time collaboration and timely, coordinated patient care. Furthermore, communication remains a crucial aspect of any multidisciplinary model. Effective communication, led by chief experts, remains critical for both models [30], and continuous improvement of these mechanisms is necessary [31].

Regarding the scope of application of these two models, based on physician insights and patient satisfaction data, we can reasonably anticipate their future application. The classical MDT meeting remains essential for all oncology departments, ensuring comprehensive discussion and physician education. For regional hubs featuring the necessary resources, the establishment of a multidisciplinary clinic model for diagnosis and treatment is highly encouraged. This model has the potential to attract patients from surrounding areas, particularly those seeking specialized and comprehensive care. Finally, in terms of the tumor types addressed at MDT clinics, the multidisciplinary clinic model is viewed as suitable for more complex or rare tumor types. The European Cancer Organisation has recently published a collection of articles discussing “essential prerequisites for delivering high‐quality cancer care,” emphasizing that a multidisciplinary approach is imperative for certain tumor types, particularly those that are rare [32]. By aligning tumor complexity with the hospital's specialized services, patients can benefit from more targeted and comprehensive care.

This study has several limitations. First, as a single‐center study conducted in a leading tertiary cancer hospital, the findings may not be fully generalizable to community hospitals or healthcare settings with different resource capacities. Second, the focus on a single tumor center limits the exploration of model applicability across a broader spectrum of diseases. Finally, the cross‐sectional design of the patient satisfaction survey captures perceptions at a single point in time. Future research should validate the MDT clinic model's effectiveness in other tertiary centers, adapt its framework for use in secondary hospitals, and adopt longitudinal designs to investigate long‐term clinical outcomes and cost‐effectiveness. Expanding this comparative approach to general hospitals and non‐oncological diseases would further test its transferability.

## 5 Conclusions

In conclusion, both the classical MDT meeting model and the MDT clinic model exhibit unique strengths and applications. Hospitals can employ the classical MDT meeting model to facilitate in‐depth discussions and learning opportunities, while the MDT clinic model can be used to advance patient‐centered care and improve patient satisfaction. The most important consideration in this regard is to choose the most appropriate model based on individual patient needs and the actual availability of medical resources. Furthermore, continuous improvement in communication mechanisms and clinical workflows is critical to enhancing the effectiveness of multidisciplinary care.

## Author Contribution

Conceptualization: Xiaoying Jiang and Mengying Li. Investigation: Leihua Chen and Yanbing Liu. Data curation and Formal analysis: Mengying Li and Leihua Chen. Writing ‐ original draft: Mengying Li. Writing ‐ review and editing: Xiaoying Jiang. All authors have read and approved the final version of the manuscript. Dr. Xiaoying Jiang had full access to all of the data in this study and takes complete responsibility for the integrity of the data and the accuracy of the data analysis.

## Consent

Informed consent was obtained from all the subjects who appear in the images before the photographs were taken.

## Conflicts of Interest

The authors declare no conflicts of interest.

## Transparency Statement

The lead author Xiaoying Jiang affirms that this manuscript is an honest, accurate, and transparent account of the study being reported; that no important aspects of the study have been omitted; and that any discrepancies from the study as planned (and, if relevant, registered) have been explained.

## Supporting information


**Table S1:** Annual visits of MDT clinics and MDT meetings. **Table S2:** Comparison of the MDT meeting and the MDT clinic in breast cancer in 2021.

**Supporting material 2:** Questionnaire for patients.

**Supporting material 3:** Interview outlines for physicians.

## Data Availability

The datasets generated during this study are not publicly available to protect the confidentiality and privacy of the study participants, in accordance with the ethical approval and informed consent agreements. However, aggregated data or a minimal anonymized dataset supporting the main findings are available from the corresponding author upon reasonable request for legitimate academic or research purposes.
