# Supplementary material for: A Dual‐Perspective Comparison of Classical and Clinic‐Based Multidisciplinary Team Models in Cancer Care: A Cross‐Sectional and Qualitative Study
Source: Health Sci Rep. 2026 Jan 28;9(2):e71787. doi: 10.1002/hsr2.71787 (PMC12852503; doi:10.1002/hsr2.71787)
Supplement: Supplementary file 1 — Table S1: Annual visits of MDT clinics and MDT meetings. Table S2: Comparison of the MDT meeting and the MDT clinic in breast cancer in 2021. [file HSR2-9-e71787-s003.docx]

As shown in Tables S1, in May 2020, a total of 6 MDT clinics were established for pancreatic, breast, colorectal, gynecology and urinary tumors, account for an annual outpatient volume of 2,394 people. Following the establishment of the MDT clinic, a significant decrease was observed in the number of patients discussed in classical MDT meetings during the same year. In 2021, MDT clinics for neuroendocrine, head and neck, and hepatobiliary tumors were successively established, accounting for an annual outpatient volume of nearly 6,000 people.

Table S1 Annual visits of MDT clinics and MDT meetings

| Type of tumor | MDT clinic | | | | MDT meeting | | | | |
| --- | --- | --- | --- | --- | --- | --- | --- | --- | --- |
|  | 2020 | 2021 | 2022 | Sum | 2018 | 2019 | 2020 | 2021 | 2022 |
| Pancreatic tumor | 978 | 1545 | 597 | 3120 | 180 | 188 | 3 | 0 | 5 |
| Breast tumor | 782 | 1237 | 732 | 2919 | 119 | 107 | 71 | 88 | 52 |
| Colorectal tumor | 170 | 308 | 109 | 587 | 487 | 539 | 331 | 453 | 285 |
| Gynecological tumor | 402 | 1717 | 694 | 2813 | 38 | 25 | 15 | 10 | 13 |
| Urinary tumor | 62 | 174 | 71 | 307 | 371 | 313 | 219 | 298 | 214 |
| Neuroendocrine tumor |  | 652 | 489 | 1141 |  |  |  |  |  |
| Head and neck tumors |  | 64 | 33 | 97 | 333 | 336 | 217 | 313 | 241 |
| Hepatobiliary tumor |  | 78 | 118 | 196 | 232 | 255 | 250 | 284 | 193 |
| Sum | 2394 | 5775 | 2843 | 11012 | 1760 | 1763 | 1106 | 1446 | 1003 |

*Note*. MDT = Multidisciplinary Team.

Table S2 Comparison of the MDT meeting and the MDT clinic in breast cancer in 2021

| Variables | | MDT meeting | MDT clinic |
| --- | --- | --- | --- |
| Hospitalization or not | Not hospitalized | 22(50.00) | 947(76.56) |
|  | Outpatient radiotherapy | 55(62.50) | 158(12.77) |
|  | Hospitalized | 11(12.50) | 132(10.67) |
| Subgroup Analysis of Hospitalized Patients* (MDT Meeting: n=11; MDT Clinic: n=132) | | | |
| Surgery or not | Yes | 11(100.00) | 122(92.42) |
|  | No | 0(0.00) | 10(7.58) |
| Radiotherapy or not | Yes | 6(54.55) | 32(24.24) |
|  | No | 5(45.45) | 90(68.18) |
| Average total hospital stay | | 7.18 | 5.94 |
| Average preoperative hospital stay | | 4.18 | 3.60 |

*Note*. *The following analysis of surgery, radiotherapy, and length of stay is based on the 11 hospitalized patients from the MDT meeting group and the 132 from the MDT clinic group.

MDT = Multidisciplinary Team.
