# Supplementary material for: A Dual‐Perspective Comparison of Classical and Clinic‐Based Multidisciplinary Team Models in Cancer Care: A Cross‐Sectional and Qualitative Study
Source: Health Sci Rep. 2026 Jan 28;9(2):e71787. doi: 10.1002/hsr2.71787 (PMC12852503; doi:10.1002/hsr2.71787)
Supplement: Supplementary file 2 — Supporting material 2: Questionnaire for patients. [file HSR2-9-e71787-s001.docx]

**Questionnaire for patients**

1. **Basic information**
2. What is your gender?
3. Male
4. Female
5. Your date of birth：
6. What is your marital status?
7. Unmarried
8. In marriage
9. Widowed
10. Divorce
11. Other: _______
12. What is your educational level?
13. Junior high school and below
14. High school or technical secondary school
15. Bachelor's degree or college degree
16. Postgraduate or above
17. Are you the patient or their families or friends?
18. The patient
19. Families or friends
20. **Outpatient information**
21. Your visiting department is:
22. Do you live in Shanghai regularly？
23. Yes
24. No
25. Is this your first visit to this hospital?
26. Yes
27. No
28. Is this your first visit to the MDT clinic of this hospital？
29. Yes
30. No
31. How did you learn about the MDT clinic of this hospital？
32. Recommendation from doctors
33. Online media
34. Referrals from friends or family
35. Others:
36. How did you register for this clinic？
37. Doctor of first contact in this hospital
38. The hospital's WeChat official account
39. On-site registration
40. Others:
41. Days from the appointment of the MDT clinic to the actual visit：
42. **Patient satisfaction**
43. Do you find it convenient to register for the MDT clinic in this hospital？
44. Extremely convenient
45. Quite convenient
46. Neutral
47. Hardly convenient
48. Not convenient at all
49. Did you get a definite diagnosis and treatment plan after the visit？
50. Yes
51. No（jump to Ⅲ.4）
52. Will you follow this treatment plan？
53. Yes
54. No
55. Are you satisfied with the environment of the MDT clinic？
56. Extremely satisfied
57. Quite satisfied
58. Neutral
59. Hardly satisfied
60. Not satisfied at all
61. Are you satisfied with the attitude of the clinician in the MDT clinic？
62. Extremely satisfied
63. Quite satisfied
64. Neutral
65. Hardly satisfied
66. Not satisfied at all
67. Do you think the MDT clinic can provide more benefits for your treatment？
68. Totally agree
69. Quite agree
70. Neutral
71. Hardly agree
72. Not agree at all
73. Do you think the charge for the MDT clinic is reasonable？
74. Extremely reasonable
75. Quite reasonable
76. Neutral
77. Hardly reasonable
78. Not reasonable at all
79. Would you recommend the MDT clinic to other similar patients？
80. Yes
81. No
82. Any other questions or suggestions：
