# Supplementary material for: A Dual‐Perspective Comparison of Classical and Clinic‐Based Multidisciplinary Team Models in Cancer Care: A Cross‐Sectional and Qualitative Study
Source: Health Sci Rep. 2026 Jan 28;9(2):e71787. doi: 10.1002/hsr2.71787 (PMC12852503; doi:10.1002/hsr2.71787)
Supplement: Supplementary file 3 — Supporting material 3: Interview outlines for physicians. [file HSR2-9-e71787-s002.docx]

**Interview outlines for physicians in multidisciplinary teams**

1. Basic information including sex, age, professional title, working years, core or extension team members.
2. Please briefly introduce the communication mode of your multidisciplinary team.
3. Will most discussed cases lead to definitive treatment plan？If not, what is the subsequent process？
4. Do team members have the channels to question or disagree with the team leader's decision during the case discussion？
5. Do you think participating in the multidisciplinary team is helpful for your personal development? What is the specific performance？
6. Compared with the traditional multidisciplinary conference discussion, what are the advantages and disadvantages of the MDT clinic in your opinion？
7. Some scholars believe that the development of MDT clinic may not be cost-effective because it requires more time and effort for clinicians. Do you agree with this view？
8. Would you recommend this mode to your patients and why？
9. Do you have any other needs or suggestions for the construction of multidisciplinary teams in our hospital？
